# Brace Treatment for Adolescent Idiopathic Scoliosis

**DOI:** 10.3390/jcm7060136

**Published:** 2018-06-04

**Authors:** Hiroshi Kuroki

**Affiliations:** Department of Orthopaedic Surgery, National Hospital Organization Miyazaki Higashi Hospital, Miyazaki 880-0911, Japan; hiroshik@med.miyazaki-u.ac.jp; Tel.: +81-985-56-2311

**Keywords:** adolescent idiopathic scoliosis, brace treatment, Osaka Medical College (OMC) brace, hanging total spine X-ray, genetic test

## Abstract

In the past, numerous non-operative treatments for adolescent idiopathic scoliosis (AIS), including exercise, physical therapy, electrical stimulation, and brace treatment, have been tried to delay or prevent the curve progression. Of these, brace treatment is the only option that is widely accepted and has demonstrated the efficacy to alter the natural history of AIS. Recently, the importance of brace treatment for AIS has been increasing since the efficacy was objectively established by the BrAIST (Bracing in Adolescent Idiopathic Scoliosis Trial) study in 2013. This editorial article summarizes the current status of brace treatment in patients with AIS and discusses future prospects on the basis of our clinical experiences.

## 1. Introduction

Scoliosis is a lateral curvature of the spine measuring at least 10° on an X-ray as determined by the Cobb method. Structural scoliosis is characterized by vertebral and trunk rotation [1]. Untreated cases of adolescent idiopathic scoliosis (AIS) may progress, and severe cases are at an increased risk for various morbidity problems and mortality [2]. In numerous non-operative treatments for AIS, brace treatment is the only potentially effective method in preventing curve progression and the subsequent need for surgery [3]. Various types of braces have been invented and were practically used in the past. In this editorial article, the current status and future prospects of brace treatment in patients with AIS will be summarized and discussed.

## 2. Aim of Brace Treatment

The goal of brace treatment for AIS is to halt the progression of a curve and to improve cosmetic appearance in accordance with maintaining whole body alignment and balance during a period of growth. Efficacy of brace treatment in AIS has continued to be controversial, with some authors reporting control of curve progression with bracing and others reporting that bracing fails to alter the natural history [4]. However, this is no longer true as evidence from the BrAIST (Bracing in Adolescent Idiopathic Scoliosis Trial) study [3] has established the effectiveness of bracing as early, non-operative care, that can statistically reduce the number of patients with AIS that progress to high-risk curves and the threshold for surgery [5]. In this study, 242 AIS patients were assigned to bracing and observation. Patients in the bracing group were instructed to wear the brace for at least 18 h per day. The rate of treatment success (skeletal maturity without 50 degrees of curve progression) was 72% after bracing, as compared with 48% after observation in the primary analysis. With the results of the BrAIST multicenter National Institute of Health (NIH) trial, there is level I evidence to support the efficacy of brace treatment in AIS [6].

## 3. History of Brace Treatment

The history of brace treatment began in the 16th century when Paré advocated metal braces made by an armorer for scoliosis; however, these braces were not used generally [7]. Before this, traction was a popular technique for correcting spinal deformities since Hippocrates’ time. In 1946, Blount and Schmidt theoretically developed the Milwaukee brace (CTLSO: cervico-thoraco-lumbo-sacro orthosis) for postoperative poliomyelitis and subsequently adopted it for treatment of AIS [8]. After that, brace treatment became popular, and various different thoraco-lumbo-sacro orthoses (TLSOs) were designed and practically utilized in place of CTLSO, which has some shortcomings; such as conspicuous design, difficulty to wear, compression of the mandible, and so on.

## 4. Type of Brace

**Figure 1:**
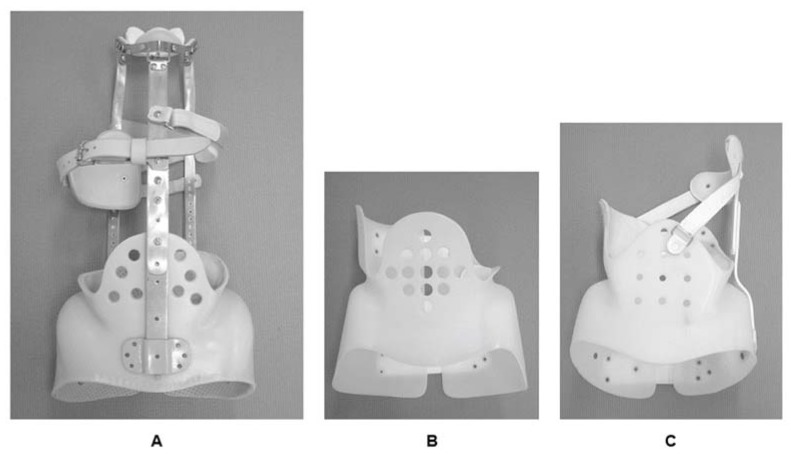
Various types of brace. (**A**) CTLSO (Milwaukee brace); (**B**) TLSO (Boston brace); (**C**) TLSO (Osaka Medical College: OMC brace).

Braces employed for the treatment of spinal deformity are divided into CTLSO and TLSO. They are implemented depending on the location of curves, that is, the CTLSO is appropriate for the upper thoracic curve, where its apex is over T7 and would be difficult to correct using the TLSO. However, patient acceptance is poorer with CTLSO because of the visible neck ring [7]. The only CTLSO is the Milwaukee brace (Figure 1A). On the other hand, various different TLSOs have been manufactured around the world, for example: Boston (Figure 1B), Wilmington, Providence, and Rosenberg braces in North America, Chêneau and Sforzesco braces in Europe, and OMC (Osaka Medical College) brace (Figure 1C), CBH (Chiba brace high type), and TLSO Hiroshima in Japan. One of the specific TLSOs is the Charleston bending brace. This brace is worn on the inside of the bending position of the convex side during only sleeping hours as a night brace in expectation of excessive correction.

## 5. Theory of Curve Correction

**Figure 2:**
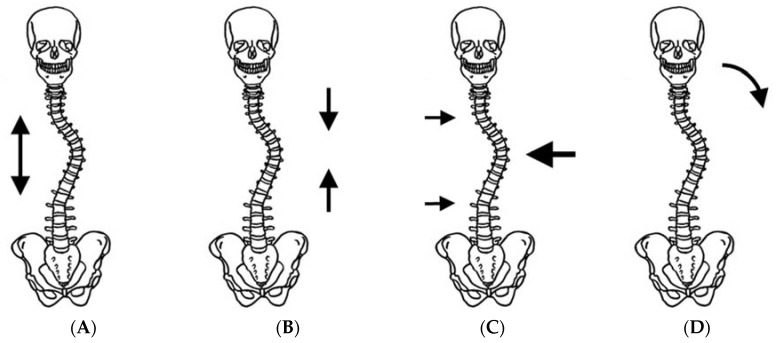
Mechanical forces to correct spinal deformity. (**A**) Distraction force; (**B**) compression force; (**C**) transverse force; and (**D**) bending force.

**Figure 3:**
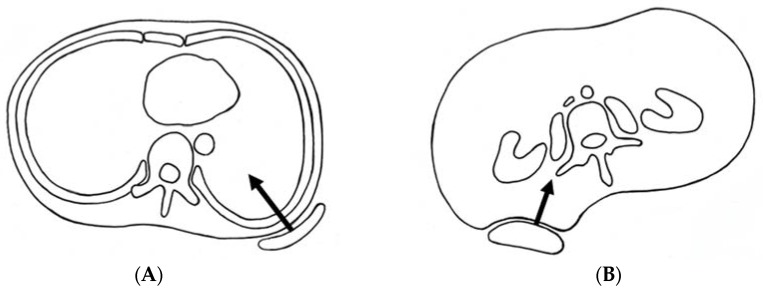
Optimal position of pads to add proper mechanical forces. (**A**) Thoracic spine; (**B**) lumbar spine.

The main mechanical forces to correct spinal deformity consist of distraction forces on the concave side, compression forces on the convex side, transverse forces from both sides, and side bending for the convex side (Figure 2). Of these, longitudinal forces are most efficient for larger curves. However, transverse forces at the apex of the scoliotic curve are more efficient than longitudinal forces when correcting a spinal deformity of less than approximately 50°, which is the common indicator for brace treatment [9]. Basically, longitudinal forces cannot be applied for patients in TLSO. However, a biomechanical study proved that 2.5 times (about 5 kg) longitudinal forces could be created by CTLSO in the recumbent position compared with an upright position [10]. Only CTLSO can control the upper thoracic curve at its apex when over T7. Whereas, the active correction of the upper thoracic curve by righting reflex may be expected with TLSO. The position of the pads is also very important to properly correct spinal deformity, especially in cases with over a 40° curvature, because deformity of the thoracic cage may deteriorate from compression forces. In such cases, angles between the vertebral column and the ribs at the apex are ordinarily acute. If the thoracic pad comes closer to the midline, the anterior force is increased, and it will facilitate thoracic hypokyphosis that originally exists in AIS. If the thoracic pad is in a lateral position, the straight lateral force may further rotate the spine in an undesirable direction. Therefore, the location of the pad should be adjusted meticulously to provide optimal anterior and transverse forces in the thoracic spine (Figure 3A). Similarly, in the lumbar spine, the lumbar pad should be located at the level of the apex to push the transverse process from the posterolateral direction and create bending and derotational forces (Figure 3B). To do so, reduction of lumbar lordosis in brace wear is absolutely required.

## 6. Complications

There exist some potential complications and problems in brace treatment. They consist of two major elements, that is, physical changes by compression of the body and psychological disturbance by the appearance of wearing a brace. The onset of a pressure sore, skin color change, and cutaneous nerve involvement are common side effects in most patients during brace treatment. Prolonged TLSO wearing may produce a tubular thorax deformity [7]. The temporomandibular joint disorder by the mandibular pad was a serious issue of the original Milwaukee brace. Reflex esophagitis due to increased intragastric pressure; decrease in glomerular filtration rate and total lung capacity has also been noted [7]. These physical changes by compression of the body trunk can be mostly controlled by meticulous modifications of the brace and skin hygiene. Whereas, management of psychological disturbance by the appearance of brace wearing is extremely difficult. Matsunaga et al. [11] reported that the rate of patients with psychological problems increased from 7.6% to 82.1% one month after the start of brace treatment. MacLean et al. [12] mentioned the psychological effects of brace treatment for, not only the patients themselves, but also their parents.

It is important to provide the patients, their parents, and nursing teachers with a better understanding of the significance of brace treatment. Further, it is needless to say that the emotional stress during brace treatment should be relieved as much as possible by mental support for the patients with brace treatment with frequent and periodic consultation.

## 7. Brace Management Protocol

We usually use the OMC brace for the treatment of AIS. The OMC brace is one of the popular custom-made TLSOs in Japan and was developed by Onomura in the 1970s [13]. The characteristics of the OMC brace are represented by its inconspicuous design, lightweight, reduction of restriction on chest wall movement, and ability to correct the high thoracic curve by righting reflex [13]. The concept of this brace is the maintenance of the whole body alignment and balance. For the achievement of these goals, step-by-step molding from pelvic girdle to high thoracic level with correcting lumbar and main thoracic curves is important to generate desirable mechanical force based on the principle of three points lateral compression.

We prescribe the OMC brace to patients who meet certain requirements as follows; still growing, a Cobb angle of between 25 and 50°, and an apex of caudad to T7, with the expectation to halt the progression of the curve and to improve cosmetic appearance. However, we have practically recommended brace treatment for immature (premenarche) AIS patients with Cobb angle of between 20 and 25° in accordance with the principles of Weinstein et al. [14].

All OMC braces were fabricated by the certified orthotist. Each OMC brace was customized for the patient from a molding box, which was directly created relative to each patient’s body. The appropriate application and fit of the brace was confirmed to ensure an accurate reflection bringing forces that were employed to reduce the size of the curve in each patient. In addition, standing anteroposterior and lateral spine X-rays were used to document the amount of curve correction and maintenance of the preferable spinal alignment while the brace was being worn. It is indisputable that extra consideration should be given to brace wear comfort and secure decompression of the bone prominences.

Patients were instructed to wear the brace for a minimum of 20 h per day at the beginning of brace treatment. We periodically followed up with patients every three to eight months depending on the maturity of the patient, to document any adverse events during brace treatment (posture, skin trouble, breakage of brace) in addition to brace wear compliance (actual brace wear time). When skeletal maturity was noted, that is, all of the following three criteria were fulfilled; a Risser stage of four, at least two years passed since the onset of menstruation (for girls), two consecutive visits over a time period of at least one year with no more than a 1-cm increase in height, brace weaning was started and advanced step by step during one year. Then, the patients were weaned off the brace a year after skeletal maturity. During brace treatment, we always spent substantial time trying to recognize the patients that brace treatment was effective but required daily effort for long periods of time.

We found that compliance of brace wearing had a tendency to diminish with time, especially in periods when changes in environment, such as the time proceeding the next stage of the education process, as an example [15]. Therefore, encouragement of patients at this period is important to maintain brace wearing as scheduled.

**Figure 4:**
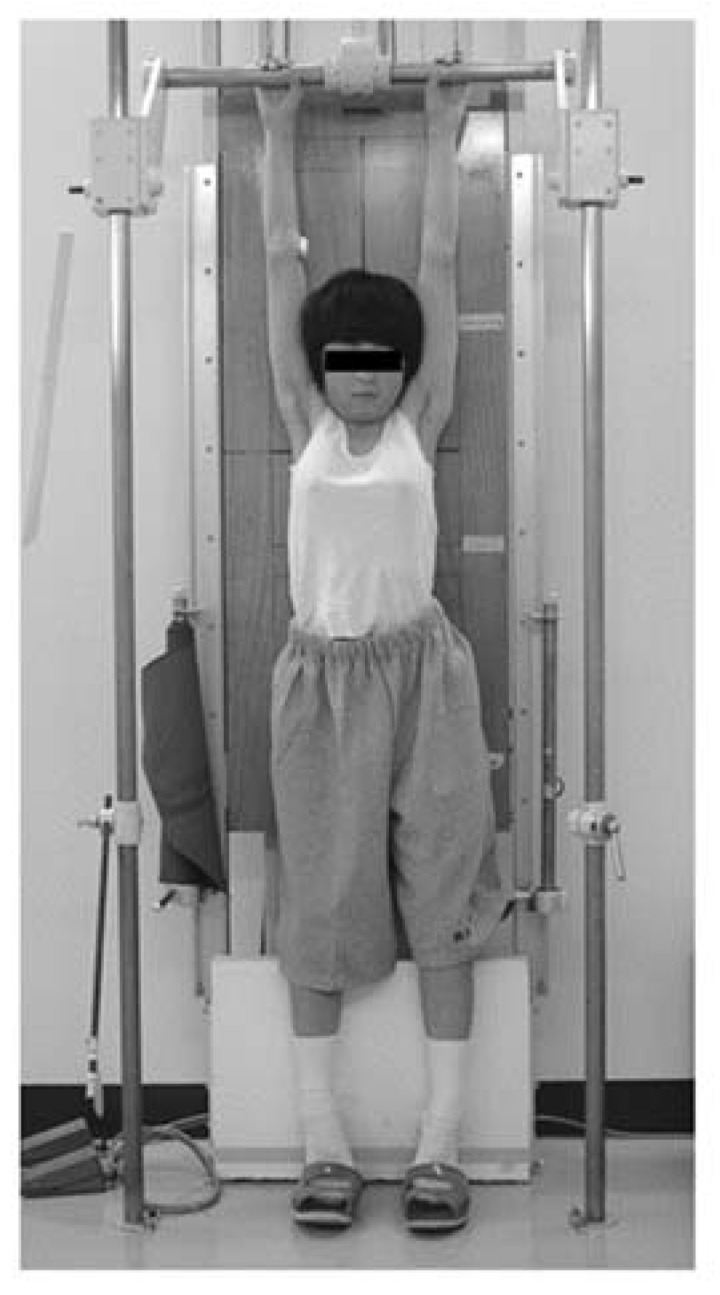
Position of hanging total spine X-ray. Hanging total spine X-ray was taken in a position that the patient is hanging onto the bar, stretching the back, and touching the toes lightly to the floor, not to sway the body under the instruction of making a great effort to stretch their back as much as possible [16] (licensed under CC BY).

The response of scoliotic deformity to initial brace wearing is essential to determine positive outcomes of brace treatment. The initial in-brace corrections are commonly different among patients due to spinal flexibility and curve patterns. Until now, various types of stress radiographs to evaluate spinal flexibility in patients with AIS have been reported; supine position, prone position, and lateral bending position. We evaluated the flexibility of the spine in patients with AIS by hanging total spine X-ray before the OMC brace treatment and assess if an appropriate correction by brace is achieved (Figure 4) [16]. This radiograph is easily taken in the outpatient clinic without any expensive equipment, extra-time, and extra-workforce. Cobb angles in hanging position were closely correlated with those on initial brace wearing, independent of curve patterns, except some curves in multiple curve patterns, and were useful for the confirmation of adequate correction by the brace. We basically aim smaller Cobb angles on initial brace wear than those in hanging position, particularly in immature patients.

## 8. Clinical Results of Brace Treatment (under SRS Criteria)

The efficacy of brace treatment for AIS continues to be controversial by reasons of the lack of consistency of both the inclusion criteria of subjects and the definition of brace effectiveness. To make the comparison among studies more valid and reliable, the Scoliosis Research Society (SRS) has standardized criteria for brace studies in patients with AIS. The SRS criteria consist of; age is 10 years or older when the brace is prescribed, Risser 0–2, primary curve angles 25–40°, no prior treatment, and if female, either premenarche or less than one year postmenarchal [17].

We previously attempted the clinical study to evaluate the efficacy of OMC brace for AIS in accordance with the modified SRS criteria (immature AIS patients who have a progressive curve from 20 to 24° were included as subjects) and compared our results with other previous reports.

In our previous study [18], 67.7% of patients achieved curve progression of less than 6° at skeletal maturity. Further, only 9.7% of patients reached Cobb angle of more than 45° which meant surgical indication. These results verified that OMC brace treatment could change the natural history of AIS just like other TLSOs [3,14,18,19,20,21,22,23,24,25,26] (Table 1). With regard to the ability of curve correction, the average initial in-brace correction of the OMC brace was 46.8%. This was inferior to the Charleston bending brace but almost the same as the other TLSOs [18,26,27,28,29,30,31,32,33,34,35,36,37] (Table 2). As previously mentioned, although the OMC brace is a TLSO, correction and controlling of the upper thoracic curve in double thoracic scoliosis could be achieved by utilizing the righting reflex that was generated by the active bending for the high thoracic curve under bracing [13].

## 9. Future Prospects

Our clinical experiences identified that maintenance of compliance, avoidance of dropout, and support for emotional burden were essential factors during brace treatments. In particular, maintenance of compliance was directly associated with clinical results. However, in brace treatment, there exists an inevitable issue of over-treatment for AIS patients, who may be free from the possibility of progress. Recently, prediction of curve progression has been made possible by the advancement of genetic testing [38,39]. Bohl et al. [40] reported that a genetic test with Scoliscore could anticipate Providence brace success. This problem may be solved by categorizing AIS patients according to the level of potential for deterioration, utilizing genetic diagnosis, so that the most appropriate treatment can be provided to each AIS patient.

## 10. Summary

Brace treatment is indispensable for AIS management as conservative care because it can alter the natural history of AIS and significantly decrease the progression of curve in skeletally-immature patients. However, it is not easy to smoothly accomplish brace treatment, since brace wearing must be, not only physically, but also emotionally, burdensome for adolescent patients. In the future, not only the development or improvement of more effective braces, and reinforcement of patient support, but also the introduction of a tailor-made treatment in which each patient can utilize genetic diagnosis and are expected to maintain motivation for brace wearing and to avoid dropouts relative to treatment success.

## Figures and Tables

**Table 1:** Literature review of the clinical results under SRS criteria.

Author (Year)	Treatment Period	Type of Brace	Success Rate	Progression Rate for Surgical Indication
Coillard C et al. (2007) [19]	?	SpineCor Brace	89.4% **	24.1% ^†^
Janicki JA et al. (2007) [20]	1y5m	TLSO	85.4% *	62.4% ^†^
	1y4m	Providence	68.6% *	42.9% ^†^
Negrini S et al. (2009) [21]	4y2m	Lyon, SPoRT	95.8% **	0.0% ^†^
Aulisa AG et al. (2009) [12]	4y11m	Progressive Action Short Brace	100% *	0.0% ^†^
Zaborowaka-Sapeta K et al. (2011) [23]	2y8m	Chêneau brace	48.1% *	12.7% ^‡^
Lee CS et al. (2012) [24]	2y9m	Charleston Bending Brace	84.2% **	12.6% ^†^
Weinstein SL et al. (2013) [3,14]	?	TLSO	—	28.1% ^‡^
Maruyama T et al. (2013) [25]	1y9m	Rigo-Chêneau brace	70.8% **	18.2% ^†^
Yamazaki K et al. (2013) [26]	6y5m	Under Arm Brace	59% **	13.6% ^†^
Kuroki H et al. (2015) [18]	3y4m	Osaka Medical College Brace	67.7% **	9.7% ^†^

y, year; m, month; ?, unknown; * Progress < 5°; ** Progression < 6°; ^†^ Progression ≥ 45°; ^‡^ Progression ≥ 50°.

**Table 2:** Literature review of the initial correction rate.

Author (Year)	Apex	Type of Brace	Correction Rate
Watts HG et al. (1977) [27]	below T10	Boston Brace	54.7%
Uden A et al. (1982) [28]	below T7	Boston Brace	41.0%
		Milwaukee Brace	10.0%
Jonasson-Rajala E et al. (1984) [29]	below T8	Boston Thoracic Brace	46.2%
		Boston Milwaukee Brace	29.3%
		Boston Brace	36.9%
Ohta K et al. (1988) [30]	—	Active Corrective Brace	53.8%
Kawakami N et al. (1991) [31]	—	Active Corrective Brace	17.6%
Asazuma T et al. (1991) [32]	below T7	Under Arm Brace	23.0% *
Arai S et al. (1992) [33]	—	Milwaukee Brace	44.2% *
Iwaya D et al. (1997) [34]	below T7	Charleston Bending Brace	75.0%
Semoto Y et al. (1999) [35]	below T7	OMC Brace	35.5%
Spoonamore MJ et al. (2001) [36]	—	Rosenberger Brace	30.0%
D’Amato CR et al. (2004) [37]	—	Providence Brace	96.0%
Yamazaka K et al. (2013) [26]	—	Under Arm Brace	38.7%
Kuroki H et al. (2015) [18]	below T8	OMC Brace	46.8%

* Maximum Correction Rate.
